# A correction procedure for secondary scattering contributions from windows in small-angle X-ray scattering and ultra-small-angle X-ray scattering

**DOI:** 10.1107/S1600576724001997

**Published:** 2024-03-29

**Authors:** William Chèvremont, Theyencheri Narayanan

**Affiliations:** a ESRF – The European Synchrotron, 71 Avenue des Martyrs, 38043 Grenoble, France; Universität Duisburg-Essen, Germany

**Keywords:** secondary scattering, SAXS, small-angle X-ray scattering, USAXS, ultra-small-angle X-ray scattering, sample-dependent background

## Abstract

This article describes a correction procedure for the removal of indirect background contributions to measured small-angle X-ray scattering patterns. The high scattering power of a sample in the ultra-small-angle region may serve as a secondary source for a window placed in front of the detector. The resulting secondary scattering appears as a sample-dependent background in the measured pattern that cannot be directly subtracted. This is an intricate problem in measurements at ultra-low angles, which can significantly reduce the useful dynamic range of detection. Two different procedures are presented to retrieve the real scattering profile of the sample.

## Abstract 2

The high scattering power of a sample at low angles may lead to significant secondary scattering contributions in the measured small- and ultra-small-angle X-ray scattering patterns. A correction procedure is presented for the removal of the excess intensity and to improve the dynamic range of the measurement.

## 1. Introduction

Modern X-ray scattering instruments incorporating high-performance photon counting hybrid pixel array detectors in principle enable the acquisition of small-angle X-ray scattering (SAXS) data spanning a large dynamic range in intensity (Narayanan *et al.*, 2023 ▸). In a SAXS setup involving a 2D detector, the vacuum of the flight tube is often separated from the detector by an X-ray-transparent window. For a sample with high forward scattering power, the intense region of the pattern may generate measurable scattering by the window material in front of the detector. The wide-angle X-ray scattering (WAXS) generated by the vacuum window superimposes on the SAXS or ultra-SAXS (USAXS) from the sample. This secondary scattering effect can significantly reduce the useful dynamic range of the SAXS or USAXS data. Here the term secondary scattering is used to describe the re-scattering of a small fraction of X-ray photons initially scattered by the sample. This is very different from the double scattering occurring in the same sample volume, which is also referred to as secondary scattering in earlier light scattering (Kraut & Dandliker, 1955 ▸; Kerker & Matijević, 1960 ▸; Prud’homme *et al.*, 1974 ▸), X-ray (Dwiggins & Park, 1971 ▸; Wignall *et al.*, 1974 ▸) and electron (Dorset, 2003 ▸) diffraction literature.

In the present case, the secondary scattering manifests as a sample-dependent background, which cannot be easily removed by a simple subtraction procedure. Here again the sample-dependent background needs to be distinguished from that originating from incoherent scattering (Staples *et al.*, 2000 ▸). But it is somewhat analogous to the extraneous scattering discussed in small-angle neutron scattering (Rennie *et al.*, 2020 ▸; Barker *et al.*, 2021 ▸). The albedo contribution is usually negligible except from the beamstop, which is minimized by a slightly tapered profile along the beam direction such that the scattered X-rays from the sample do not satisfy the condition for specular reflection. The secondary scattering effect may go unnoticed when the dynamic range of the intensity measurement is lower than five orders of magnitude, as typically found in the conventional SAXS range. However, the effect becomes more pronounced when the intensity decays sharply as in the case of Porod behavior often encountered in the USAXS region. For fractal-like slower decay of intensity, the secondary scattering may be submerged beneath the sample scattering. Identifying this non-subtractable contribution and correcting appropriately is essential for a quantitative interpretation of the measured data.

A practical approach to circumvent the secondary scattering contribution is to perform a second measurement with the intense region of the 2D scattering pattern blocked or attenuated by a large beamstop (Narayanan *et al.*, 2022 ▸). An appropriate merging of the two data sets yields the correct intensity profile down to the level unaffected by the secondary scattering in the second measurement. This approach will be sufficient for the vast majority of cases involving static measurements. However, there is a disadvantage for time-resolved studies or when the sample is susceptible to radiation damage. As a result, an analytical correction procedure involving only a single measurement is desired.

This article presents a convolution procedure by which the secondary scattering contribution in the measured 2D pattern can be estimated. Then the excess contribution can be subtracted in two dimensions prior to intensity normalization. This approach yields satisfactory results down to the level allowed by the noise in the data. A good agreement is obtained with the practical approach that involves the physical masking of the intense region.

## 2. Materials and methods

### 2.1. Model systems

In order to illustrate the different aspects of the secondary scattering effect, samples having strong forward scattering that decays rapidly are required. Therefore, colloidal suspensions consisting of relatively large particles with a narrow size distribution were chosen. The first sample consisted of spherical polystyrene (PS) particles of mean radius *R*_S_ ≃ 1012 nm and a polydispersity of 0.4%. These latex particles were suspended in a solvent mixture composed of ethanol and water in 3:1 volume ratio, and the colloid volume fraction (ϕ_S_) was of the order of 0.01. The second sample involved dilute spherical silica particles with *R*_S_ ≃ 300 nm and polydispersity 1.8% suspended in water with ϕ_S_ ≃ 0.005. The third sample consisted of a dense suspension of poly(methyl methacrylate) (PMMA) particles with *R*_S_ ≃ 380 nm and a polydispersity of 5.3% suspended in *cis*-decalin with ϕ_S_ ≃ 0.5. In this case, the particles are stabilized by a steric layer composed of poly(12-hydroxystearic acid), while the other two suspensions are charge stabilized (Ottewill, 2002 ▸). All three suspensions and corresponding solvents (background) were contained in thin-walled quartz capillaries of approximately 1 mm diameter. Finally, to demonstrate the case of a slowly decaying scattering function, a thermoreversible colloidal gel sample consisting of stearyl silica particles (*R*_S_ ≃ 67 nm and polydispersity 9%) in *n*-dodecane (Sztucki *et al.*, 2006 ▸) was used. The X-ray window materials used for *ex situ* WAXS measurements were fibrous carbon, beryllium and Lexan (polycarbonate) with thicknesses of 0.4, 0.5 and 0.25 mm, respectively. The first composite con­sisted of three laminations of oriented carbon fibers bound together by a polymer epoxy (PEP Compositec). The beryllium window material was polycrystalline metal (BRUSH-WELLMAN Inc.) while the Lexan (GE) sample was amorphous polymer.

### 2.2. X-ray scattering

All X-ray scattering measurements presented here were carried out on the time-resolved ultra-small-angle X-ray scattering (TRUSAXS) instrument (beamline ID02) at the ESRF (Narayanan *et al.*, 2022 ▸). The working energy was 12.230 keV, corresponding to an X-ray wavelength (λ) of 0.1014 nm. The SAXS/USAXS patterns were recorded using an EIGER2 4M (Dectris AG) hybrid pixel array photon counting detector placed 31 m from the sample. Additional WAXS measurements were performed using a Rayonix LX170-HS fiberoptically coupled CCD detector. Measured scattering patterns were normalized to an absolute intensity scale and azimuthally averaged following the standard procedure described elsewhere (Narayanan *et al.*, 2022 ▸). The azimuthally averaged scattering profiles are presented as a function of the modulus of the scattering vector, *q*, given by *q* = (4π/λ)sin(θ/2), with θ the scattering angle.

The main technical feature of the SAXS/USAXS setup is an evacuated flight tube of length 34 m and diameter 2 m. The detectors are enclosed in a motorized wagon that travels along a rail system from one end of the tube to about 31 m from the sample position. Inside, the wagon is at atmospheric pressure, and an X-ray-transparent window of thickness 0.4 mm made of fibrous carbon composite separates the vacuum in the flight tube (Van Vaerenbergh *et al.*, 2016 ▸). The window is glued onto a stainless steel flange that can be bolted on either the inside or outside of the stainless steel front wall (thickness 30 mm) of the wagon. As a result, the distance between the center of the window and the detector can be varied from about 30 to 60 mm, depending on whether the window is mounted on the inner or outer wall of the wagon. The primary and secondary beamstops are installed in front of the fibrous carbon window (Van Vaerenbergh *et al.*, 2016 ▸).

### 2.3. Origin of the secondary scattering

In the present case, the origin of the secondary scattering is WAXS from the fibrous carbon window. Although the direct beam is blocked by a beamstop, the intense region of the scattering pattern serves as a secondary source. The secondary scattering is inherent when a window is present anywhere in front of the detector and it becomes detectable when the scattering profile decays sharply as in the case of Porod behavior (*q* ^−4^). The low-angle instrument background and associated secondary scattering can be subtracted out, but the secondary scattering originating from the strong sample scattering manifests as a sample-dependent background that cannot be measured independently and deducted.

On the TRUSAXS instrument, the secondary scattering effect becomes visible below 10^−5^ of the maximum intensity when the fibrous carbon window is close to the detector (30 mm) (Narayanan *et al.*, 2022 ▸). It may even be observable below 3 × 10^−4^ when the window is further at 60 mm from the detector, thereby limiting the useful dynamic range of the measurement. As a result, a correction procedure becomes essential for high-dynamic-range SAXS/USAXS measurements, which is described in the following section.

## 3. Results and discussion

### 3.1. WAXS from window materials

**Figure 1:**
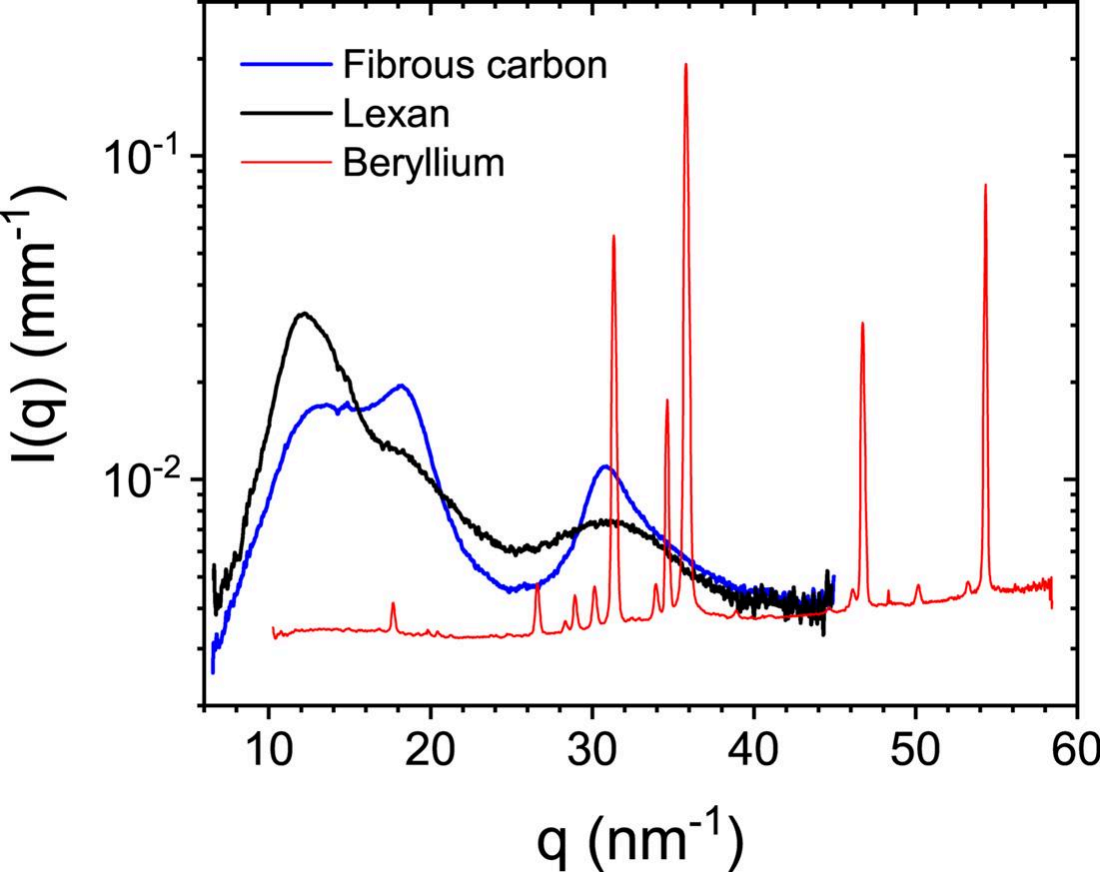
Normalized WAXS profiles of three window materials, fibrous carbon, beryllium and Lexan. The fibrous carbon 2D pattern displays oriented features, which are smoothed out in the 1D profile.

In order to illustrate the typical level of scattering by different window materials, Fig. 1 ▸ presents the normalized, azimuthally averaged WAXS profiles of the three different materials. While composite and polymeric materials have significant scattering at medium angles, beryllium has sharper diffraction rings at higher angles. As a result, the secondary scattering will be manifested earlier with the former window materials. Due to the layered architecture of the fibrous carbon window, the intensity of the WAXS profile varies up to 30% from spot to spot when measured with a small beam cross section. However, the secondary scattering is contributed by a larger area of the window of several centimetres in size and therefore the finer spatial inhomogeneity is averaged out. The secondary scattering contribution can be diminished by thinning the material, but that increases the risk of implosion of the window and consequent damage to the detector. Therefore, an optimally thick window is necessary for safe operation of the instrument and to obtain stable secondary scattering.

### 3.2. Calibration and correction procedures

**Figure 2:**
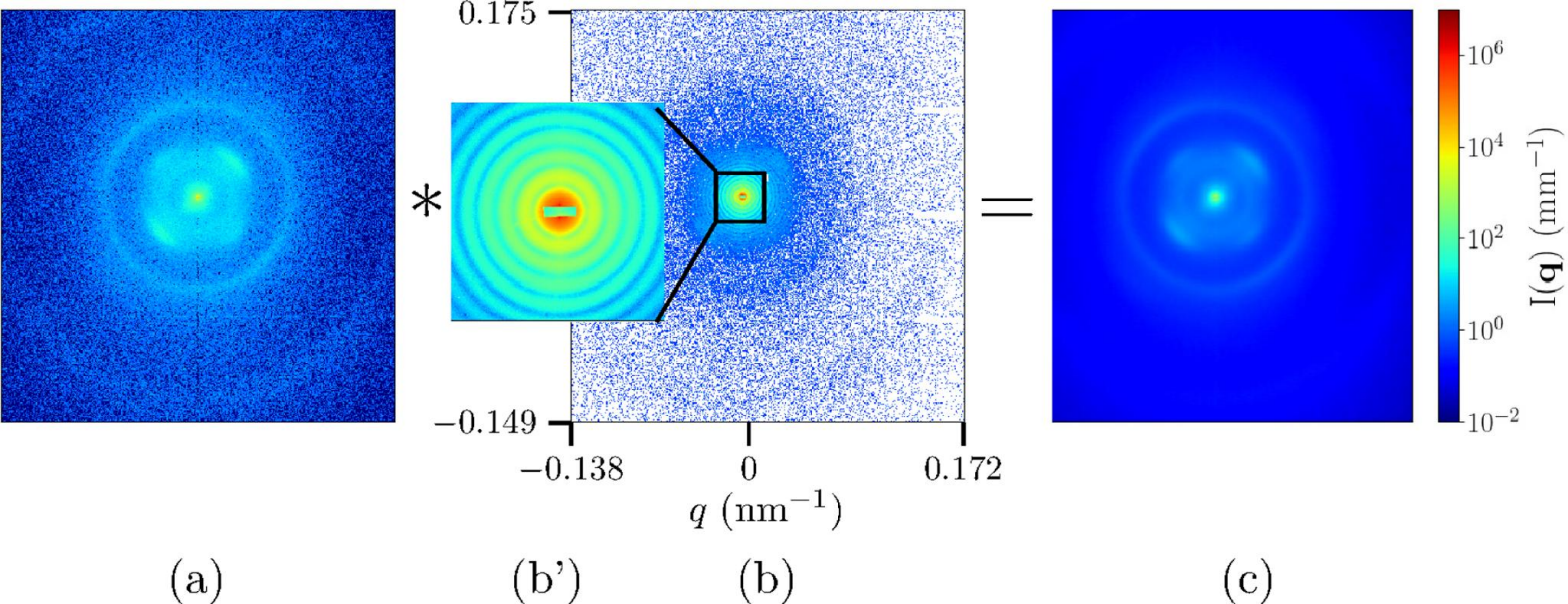
Illustration of the steps involved in the estimation of secondary scattering generated by a window in front of the detector. (*a*) Normalized WAXS pattern of the fibrous carbon window measured *in situ*, as described in the text. The intensity has been multiplied by a factor 10^9^ to display in the same color scale as the sample pattern. (*b*) Patched USAXS pattern of a dilute suspension of 2 µm-diameter PS particles recorded using the 1 × 3 mm beamstop. The inset (*b*′) depicts the intense central part of the pattern used for the convolution. (*c*) Result of the convolution of (*a*) by (*b*′), equivalently the secondary scattering contribution in (*b*).

For the application of secondary scattering correction, *ex situ* measurements as presented in Fig. 1 ▸ are not adequate since the orientation and deformation due to differential pressure need to be taken into account. Therefore, a 2D WAXS pattern of the window with sufficient statistics was recorded on the SAXS/USAXS detector (EIGER2 4M) using a highly attenuated (by a factor 10^5^) beam without the beamstop. The measured pattern was normalized by the incident beam intensity and detector response function (flat-field), and the gaps between the modules were patched using 180° rotational symmetry with respect to the center (Sztucki, 2021 ▸). The resulting pattern is shown in Fig. 2 ▸(*a*). This reconstructed pattern closely resembles the oriented diffraction diagram of carbon fiber composites (Czapski *et al.*, 2023 ▸).

Once the WAXS pattern of the window under the normal measurement condition is calibrated, the secondary scattering contribution can be estimated by the 2D convolution of this WAXS diagram by the intense region of the given scattering pattern. This operation is performed by the oaconvolve function of the *scipy.signal* package (SciPy, 2024 ▸), which involves an overlap-add method (Lyons, 2011 ▸). The convolution [equation (1)] of the longest array is decomposed into a sum of smaller convolutions [equation (2)] (this approach is efficient when one array is much larger than the other): 

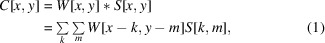




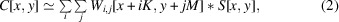




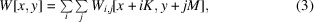

where matrices *C*[], *W*[] and *S*[] are the convoluted, window and sample scattering patterns, respectively. *W*_*i*,*j*_ are the subsets of *W* of size (*K*, *M*). *S*[] is supposed to be 0 if *x* > *K* or *y* > *M*. This implies that most of the intensity that contributes to the secondary scattering needs to be included in the subregion used for convolution. A good threshold has been found to be 10^−3^ of the maximum intensity.

Since the secondary scattering is not sample-to-detector-distance dependent, it is desirable to apply this correction prior to the conventional intensity normalization (Narayanan *et al.*, 2022 ▸). Therefore, the measured 2D patterns from the samples were only flat-field normalized and then the detector gaps were patched as before. Fig. 2 ▸ illustrates the steps from the patched 2D data to the estimation of corresponding secondary scattering in the case of a USAXS pattern from the PS colloidal suspension recorded using a 1 × 3 mm beamstop. Since the contribution strongly depends on the 2D intensity distribution, these steps need to be performed in two dimensions. The corrected pattern is obtained by the subtraction of the estimated secondary scattering from the measured pattern and then normalized by the transmitted number of photons and the solid angle subtended by the pixels (Narayanan *et al.*, 2022 ▸). The subtraction can also be done using the normalized and azimuthally averaged 1D profiles.

### 3.3. Application to colloidal suspensions

**Figure 3:**
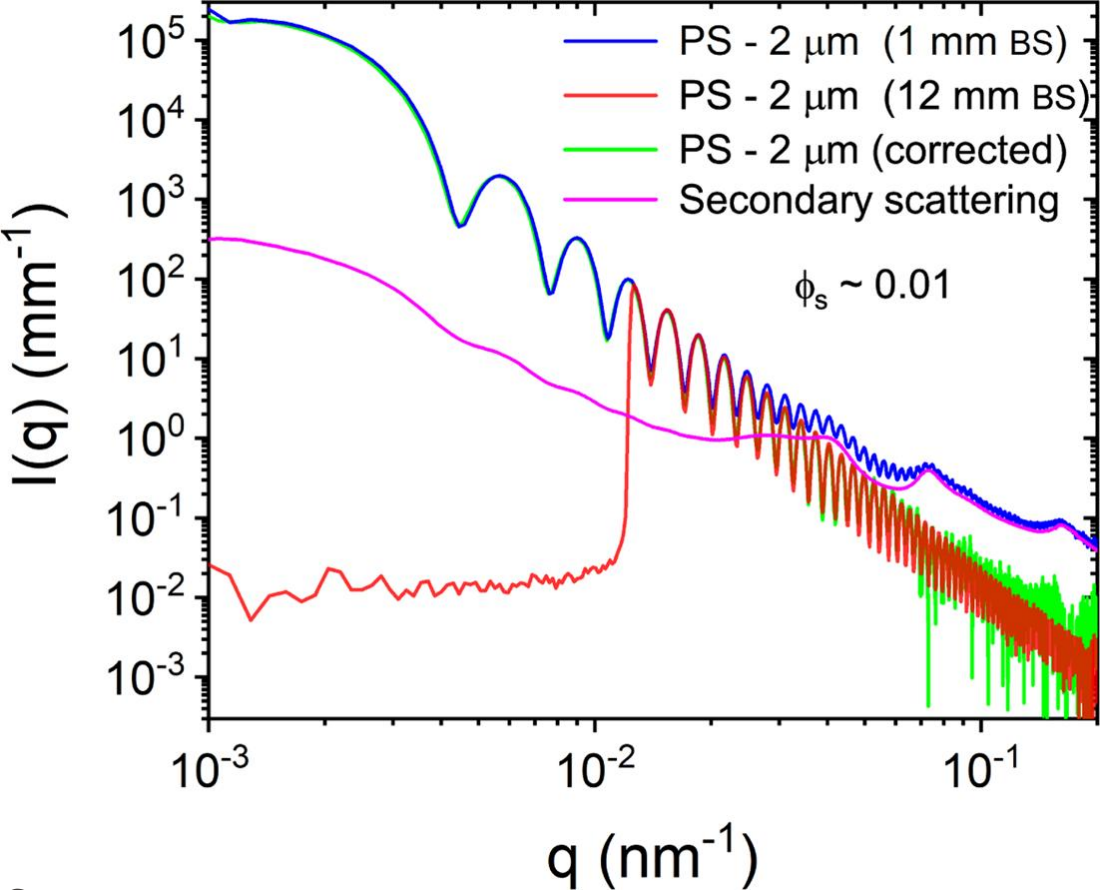
Normalized USAXS profiles of a dilute suspension of 2 µm-sized PS particles measured using a small rectangular beamstop (1 × 3 mm) and a large circular beamstop (12 mm in diameter). The former (1 mm) is strongly affected by the secondary scattering, as shown by the significant deviation at larger *q*. The estimated secondary scattering and the corrected profiles are also shown.

The USAXS from colloidal suspensions of relatively large particles is ideally suited to detect the secondary scattering effect and validate the correction procedure since the intensity profiles contain a large number of oscillations due to the spherical scattering form factor (Bessel function), the envelopes of which decay sharply as *q* ^−4^. Fig. 3 ▸ presents the normalized, azimuthally averaged and background-subtracted USAXS profiles from a dilute suspension of PS particles of approximately 2 µm diameter measured with small (1 mm) and large (12 mm) beamstops. The former is strongly affected by the secondary scattering while the latter displays the expected behavior at high *q* since the intense region of the scattering pattern is blocked by the large beamstop. The deduced secondary scattering profile shows the additional contribution to the measured intensity when using the small beamstop. Subtraction of this excess contribution from the normalized intensity yields the expected asymptotic behavior of the scattering profile.

**Figure 4:**
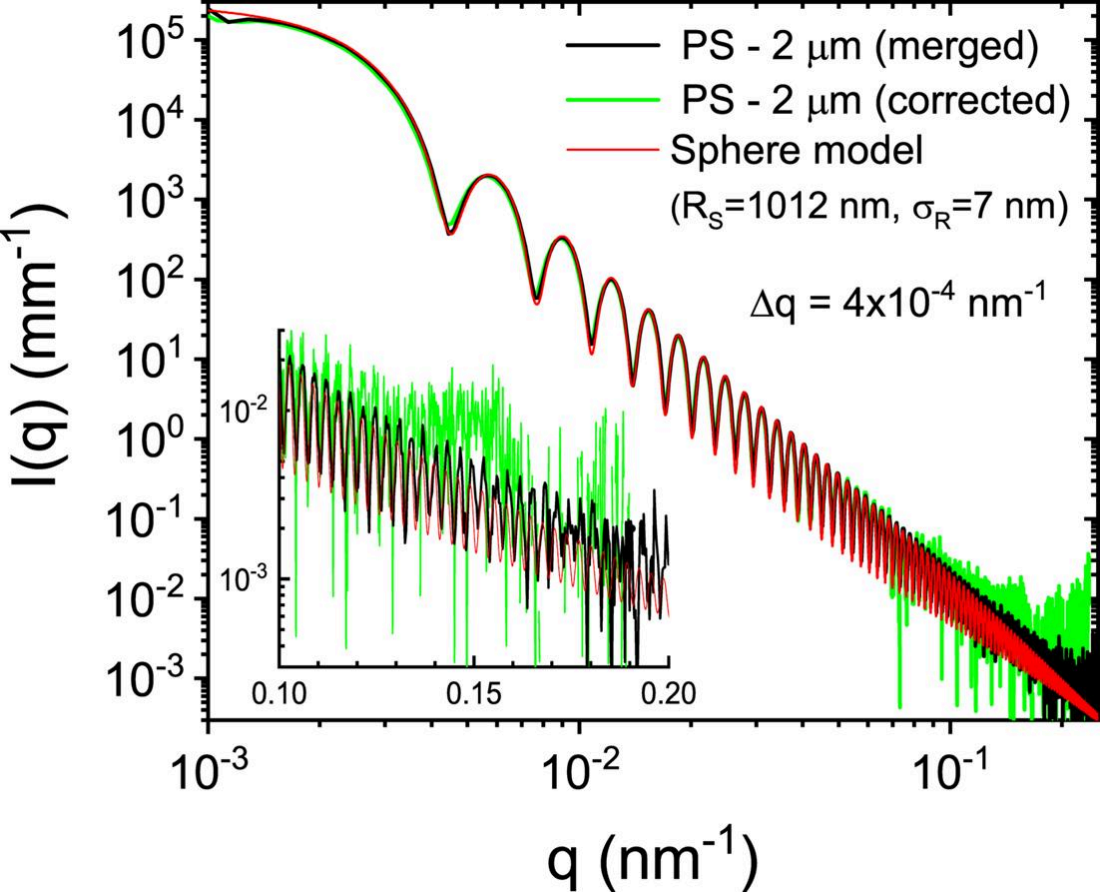
Comparison of the merged and corrected scattering profiles of PS particles with a polydisperse spherical scattering function for the parameters shown in the legend. Corresponding normalized backgrounds have been subtracted and the model has incorporated the *q* resolution (Δ*q*). The inset displays the comparison at the high-*q* region.

A more quantitative comparison of the results is provided in Fig. 4 ▸. A straightforward merging of the low-*q* region of the profile measured using the smaller beamstop with the unshadowed high-*q* section of the large-beamstop data yields the real profile. This merged profile agrees well with a model of polydisperse spherical scattering function with mean radius *R*_S_ ≃ 1012 nm and standard deviation σ_*R*_ ≃ 7 nm up to the 70th order of the spherical Bessel function. The corrected profile using the convolution procedure also closely matches the model curve. It must be recognized that the intensity statistics are much better with the merged curve as the large-beamstop profile was measured with a ten times longer acquisition time. The correction procedure enables measurement of the scattering profile in one shot with a dynamic range ∼ 10^7^, which is an advantage in time-dependent studies. To observe finer details of the scattering profile in the higher-*q* region, it is desirable to block the intense region at lower *q* and avoid the count rate limitation of the detector and associated non­linearity effect.

Additional comparisons for smaller particles, dilute silica colloids (*R*_S_ ≃ 300 nm) and a dense PMMA suspension (*R*_S_ ≃ 380 nm) are presented in the supporting information. The apparent manifestation of secondary scattering becomes less severe for smaller particles and larger polydispersities. From the estimated profile, it is clear that the secondary scattering contribution will be submerged beneath the sample scattering for *q* dependence weaker than *q* ^−2.5^. Even for *q* ^−4^ decay, the secondary scattering contribution can be hidden further by mounting the fibrous carbon window on the inner wall of the wagon, as was done before (Narayanan *et al.*, 2022 ▸). However, this has the disadvantage of an undesired heterodyning effect and reducing the apparent speckle contrast when performing X-ray photon correlation spectroscopy (Chèvremont *et al.*, 2024 ▸). As a result, the fibrous carbon window was placed furthest (60 mm) from the detector.

**Figure 5:**
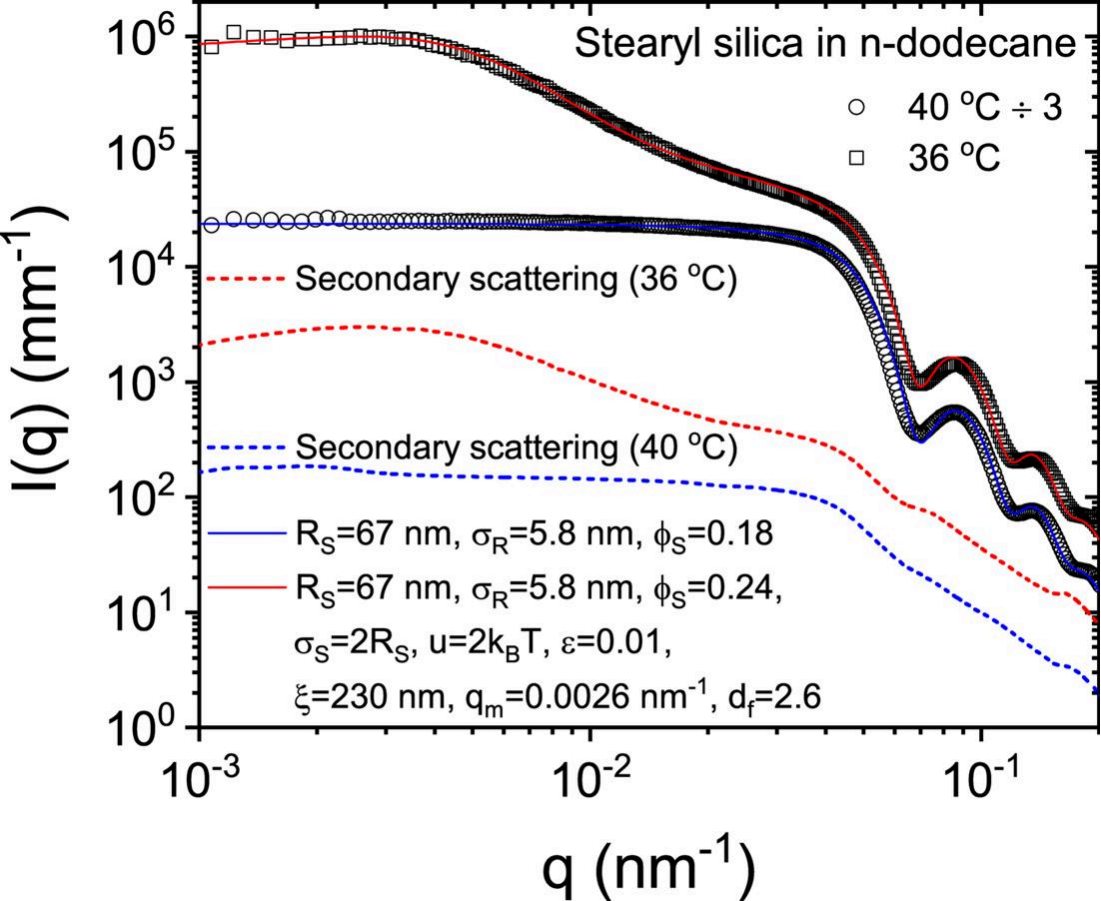
Normalized USAXS profiles from a moderately concentrated suspension of stearyl silica particles in *n*-dodecane for hard-sphere repulsive (40°C) and short-range attractive (36°C) states without the secondary scattering correction. The continuous lines are modeling using the equations given in the supporting information, with the parameters listed in the legend. The estimated secondary scattering contribution is shown by the dashed curves, which have insignificant influence on the model parameters. For clarity of presentation, the profiles for 40°C have been divided by a factor of 3.

Finally, the secondary scattering effect becomes undetectable in the case of a slowly decaying scattering function. Fig. 5 ▸ presents the normalized scattering profiles from a suspension of stearyl silica particles in *n*-dodecane at a moderate concentration (Sztucki *et al.*, 2006 ▸). This sample behaves as hard-sphere repulsive above 38°C and short-range attractive below. As a result, the sample undergoes an arrested phase separation (Zaccarelli *et al.*, 2008 ▸), displaying a spinodal-like ring upon cooling well below this temperature (Narayanan *et al.*, 2020 ▸). The continuous lines in Fig. 5 ▸ represent the modeling using the equations given in the supporting information (Narayanan *et al.*, 2020 ▸; Chen *et al.*, 2002 ▸). The estimated secondary scattering contribution is well submerged beneath the sample scattering. This is also usually the case with scattering profiles from hierarchically self-assembled systems, where each decade in *q* is fed by a particular structural feature in the sample (Landman *et al.*, 2018 ▸; Raviv *et al.*, 2023 ▸). As a result, the secondary scattering contribution is rather unimportant as shown in Fig. S5 (in the supporting information). However, when pursuing state-of-the-art SAXS modeling by means of advanced *ab initio* methods (Gräwert & Svergun, 2020 ▸; Chatzimagas & Hub, 2023 ▸; Raviv *et al.*, 2023 ▸), the secondary scattering correction can improve the accuracy of data in the higher-*q* region.

A practical implication of the secondary scattering contribution is that the merging of normalized intensities measured at two different sample-to-detector distances (approximately 30 m and 1 m) can become nontrivial. The excess intensity in the high-*q* region of the longer detector distance curtails a genuine overlap with the low-*q* region of the shorter distance measurement. The secondary scattering correction can alleviate this problem, thereby avoiding an additional measurement at an intermediate sample-to-detector distance. Another case is coherent diffractive imaging (CDI), especially involving a strongly scattering specimen (Beuvier *et al.*, 2022 ▸). Here an accurate removal of the background is critical for faithful phase retrieval and image reconstruction (Beuvier *et al.*, 2022 ▸).

In addition to WAXS, the fibrous architecture of the window also refracts the X-ray beam (Harbich *et al.*, 2001 ▸) in the ultra-small-angle region as shown in Fig. S6. However, the secondary refracted signal is nearly collected by the same detector pixel with a minor modification of the point spread function from a perfect boxcar form. The secondary USAXS scattering is more pertinent in the case of a Bonse–Hart USAXS instrument.

All window materials scatter both in the WAXS and more strongly in the USAXS range. The ideal option is to have the detector installed in vacuum without an intervening window. However, that comes with the risk of damaging the detector by shock waves in the case of an uncontrolled rupture of the vacuum.

## 4. Conclusions

A correction procedure for secondary scattering contributions emanating from a window placed between the detector and primary beamstop is presented. The correction restores the useful dynamic range of the measurement down to 10^−7^ of the maximum intensity. This procedure is applicable to both isotropic and anisotropic scattering patterns as the key operation of convolution is done in two dimensions. The method was validated using the scattering patterns from colloidal suspensions, which exhibit a large number of oscillations from the spherical form factor and whose intensity profiles decay sharply. Very good agreement is obtained between the corrected and calculated scattering profiles. This correction can improve the overlap between normalized intensity profiles measured at two different sample-to-detector distances farther apart. It will be useful to perform this correction even when the measured profiles do not directly manifest the secondary scattering contribution, especially when operations such as division of two intensities are involved, *e.g.* for deriving an experimental structure factor of interactions from the measured intensities or when performing advanced *ab initio* modeling of the SAXS data. This correction could also improve the accuracy of image reconstruction in CDI involving a strongly scattering specimen.

## 5. Data and program availability

The script demonstrating the correction procedure and the data used to create the figures in this article are available at the ESRF GitHub repository (Chèvremont, 2024 ▸).

## Supplementary Material

Supporting information containing additional examples and model used for the analysis. DOI: 10.1107/S1600576724001997/uz5008sup1.pdf
