# Supplementary material for: A correction procedure for secondary scattering contributions from windows in small-angle X-ray scattering and ultra-small-angle X-ray scattering
Source: J Appl Crystallogr. 2024 Mar 29;57(Pt 2):440–5. doi: 10.1107/S1600576724001997 (PMC11001404; doi:10.1107/S1600576724001997)
Supplement: Supplementary file 1 [file j-57-00440-sup1.pdf]

JOURNAL OF
APPLIED
CRYSTALLOGRAPHY

Volume 57 (2024)

Supporting information for article:

A correction procedure for secondary scattering contributions from windows in small-angle X-ray scattering and ultra small angle X-ray scattering

William Chèvremont and Theyencheri Narayanan

Supporting Information: A Correction Procedure for Secondary Scattering Contributions from Windows in SAXS and USAXS

William Chèvremont[a] and Theyencheri Narayanan [a]*

[a]*ESRF - The European Synchrotron, 71 Avenue des Martyrs , 38043 Grenoble, France. E-mail: narayan@esrf.fr*

1. Application of the correction procedure for different colloidal suspensions

This section presents additional examples of the application of the correction procedure for other type of colloidal suspensions with different sizes and scattering features.

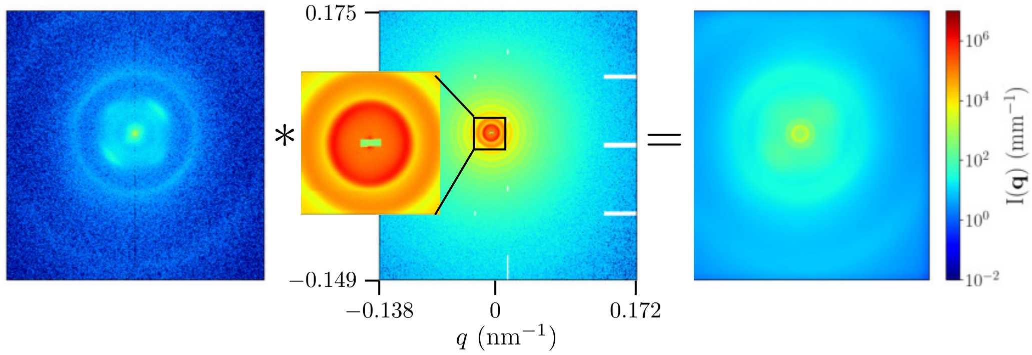

Fig. 1. Illustration of the steps involved in the estimation of secondary scattering generated by the fibrous carbon window in front of the detector. (a) Normalized WAXS pattern ($\times$ 10^9) of the fibrous carbon window measured in situ as described in the text. (b) Patched USAXS pattern of a dense suspension of 800 nm diameter PMMA particles recorded using the 1 mm $\times$ 3 mm beamstop. The inset (b') displays the intense pattern used for the convolution. (c) Result of the convolution of (a) by (b'), that is equivalent to the secondary scattering contribution in (b).

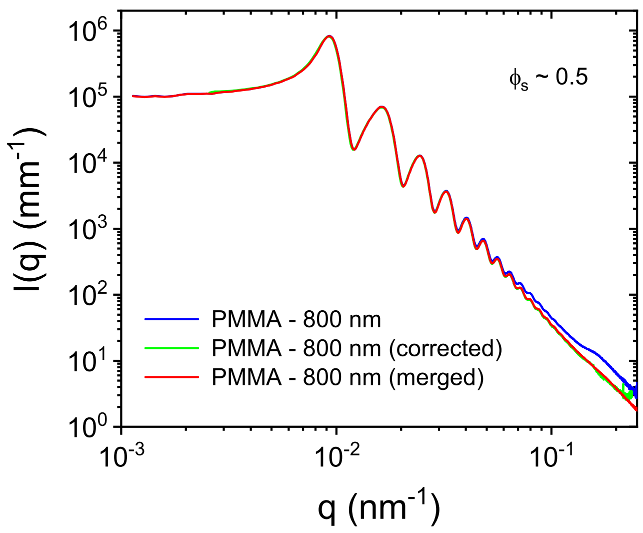

Fig. 2. Normalized USAXS profiles of the dense suspension of 800 nm sized PMMA particles without and with the correction. The good agreement between the corrected (by convolution) and merged data measured with two different beamstops (1 mm × 3 mm and 12 mm) is evident.

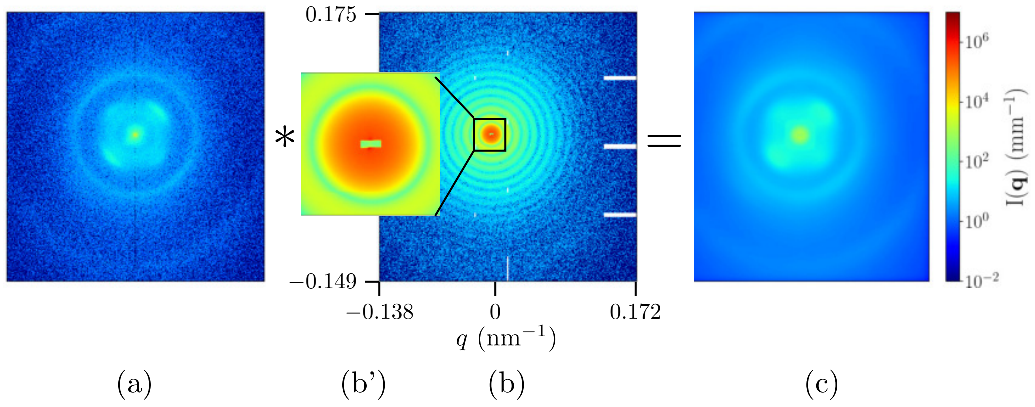

Fig. 3. Illustration of the steps involved in the estimation of secondary scattering generated by the fibrous carbon window in front of the detector. (a) Normalized WAXS pattern (× 10^9) of the fibrous carbon window measured in situ as described in the text. (b) Patched USAXS pattern of a dilute suspension of 600 nm diameter silica particles recorded using the 1 mm × 3 mm beamstop. The inset (b') displays the intense pattern used for the convolution. (c) Result of the convolution of (a) by (b'), that is equivalent the secondary scattering contribution in (b).

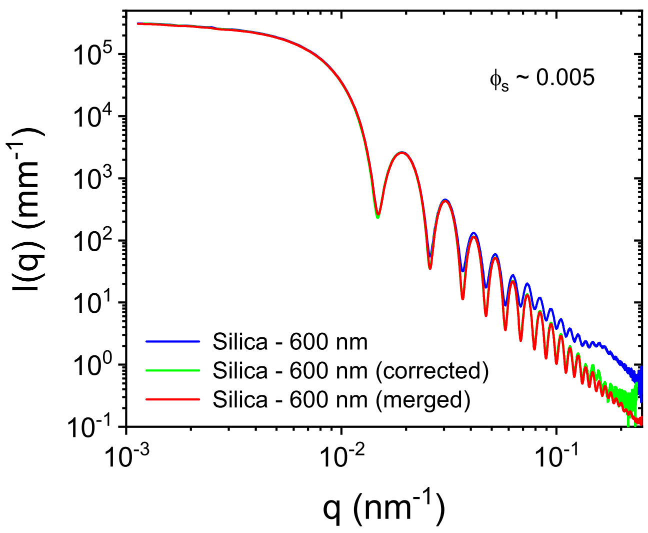

Fig. 4. Normalized USAXS profiles of the dilute suspension of 600 nm sized silica particles without and with the correction. The good agreement between the corrected (by convolution) and merged data measured with two different beamstops (1 mm × 3 mm and 12 mm) is evident.

2. Modeling of $I(q)$ data

The normalized intensity, $I(q)$, profiles from different colloidal suspensions were modeled by the following general expression (Sztucki *et al.*, 2006);

$$I(q) = N_p \Delta\rho^2 V_p^2 \langle P(q) \rangle \left[S_M(q) + S_C(q) \right] \tag{1}$$

Where N_p is the number density of particles, V_p is the mean volume of particles ($\phi_S = N_p V_p$), $\Delta\rho$ is the average contrast of particles in the solvent, $\langle P(q) \rangle$ is the scattering form factor of spherical particles averaged over their size distribution, which was assumed to be a Gaussian function. $S_M(q)$ is an effective structure factor of interaction between particles and $S_C(q)$ is the structure factor of clusters when particles aggregate due to attraction. In the case of PS sample, the suspension is dilute and particles are stabilized by electrostatic repulsion. Therefore, $S_M(q) = 1$

and $S_C(q) = 0$. For the stearyl silica particles in n-dodecane, the system is hard-sphere repulsive at higher temperatures and transforms to short-range attractive at lower temperatures. $S_M(q)$ for this system can be well-described by a square-well attractive potential of depth, u in units of k_BT (with k_B the Boltzmann constant and T the absolute temperature), and range, ϵ, relative to the hard-sphere diameter, σ_S (Sztucki *et al.*, 2006). Here $S_M(q)$ is calculated using a perturbative expansion of Percus-Yevick (PY) approximation (Chen *et al.*, 2002). For the cluster of particles, $S_C(q)$ is expressed in terms of a Lorentzian function of the following form (Narayanan *et al.*, 2020):

$$S_C(q) = \frac{I_M}{(1 + (q - q_m)^2 \xi^2)^p} \tag{2}$$

Where I_M is proportional to the mean cluster mass, $\xi = R_g/\sqrt{3p}$, with R_g the radius of gyration of clusters and the exponent, $p = d_f/2$, d_f being the fractal dimensionality of clusters, and the parameter q_m is related to the empty regions between clusters or correlation holes. At higher temperatures, when the particles are hard-sphere repulsive, $u = 0$ and $S_C(q) = 0$. When particles become attractive at lower temperatures, $u \simeq 2k_BT$ and $\epsilon \simeq 0.01\sigma_S$ (Sztucki *et al.*, 2006). With the onset of attraction, particles form clusters as manifested by strong forward scattering and a Lorentzian feature at low q. At the ϕ_S investigated, they form percolated clusters, characterized by $d_f \simeq 2.6$ (Sztucki *et al.*, 2006; Narayanan *et al.*, 2020). The parameter $2\pi/q_m$ is interpreted as the average center-center separation between clusters, which is equivalent to center-center distance of the depletion zones (Narayanan *et al.*, 2020) and $I_M = 3.9$.

3. Weak secondary scattering effect in a hierarchically organized system

For slowly decaying scattering profiles, the secondary scattering contribution is likely to be not significant. This is demonstrated using a sample consisting of sodium dodecyl sulfate (SDS) and β-cyclodextrin (CD) in 1:2 molar ratio in water (7 wt%). At

room temperature, this system forms well-defined microtubes with a multilamellar wall architecture composed of capsids of SDS with two β-CD molecules ordered in a crystalline lattice (Landman *et al.*, 2018). The intensity level of secondary scattering was evaluated using two beamstops of sizes 1 mm and 12 cm, and found to be not significant. Due to the tubular morphology and repulsive structure factor of interactions, the forward scattering at low q is very close to the background intensity and therefore the secondary scattering contribution is removed with the usual background subtraction.

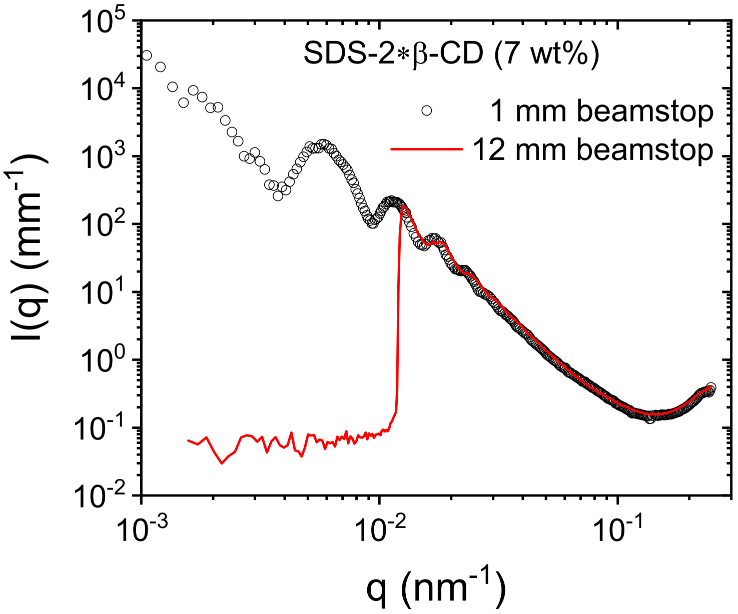

Fig. 5. Normalized and background subtracted USAXS profiles from an aqueous suspension (7 wt%) of multilamellar microtubes formed by sodium dodecyl sulfate (SDS) and β-cyclodextrin (CD) in 1:2 molar ratio at room temperature (Narayanan *et al.*, 2023). It is evident that profiles measured with the small (1 mm) and large (12 cm) beamstops perfectly superimpose at the higher q region signifying that the secondary scattering correction is unimportant in this case. The rise of $I(q)$ at the highest q is the onset of first order Bragg peak from the multilamellar organization (Landman *et al.*, 2018).

4. USAXS from the fibrous carbon window

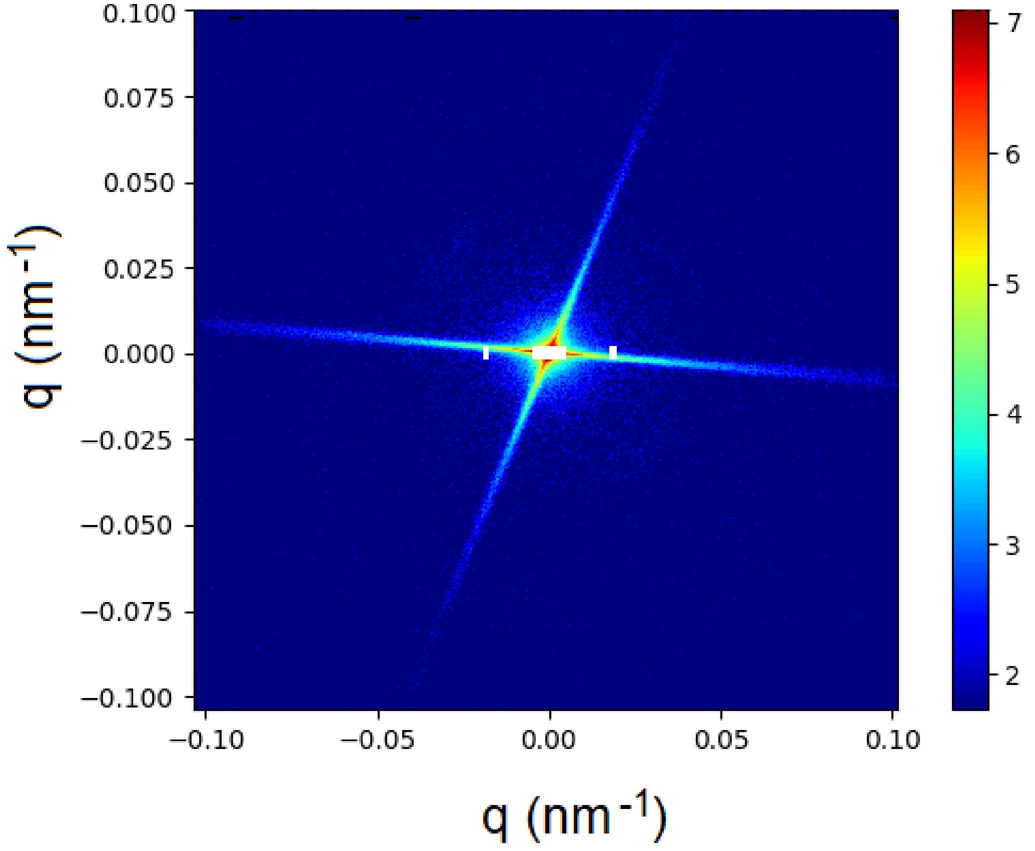

Fig. 6. Normalized USAXS pattern of the fibrous carbon window specimen indicating the orientation of the fibers within the laminations. In the first and third layer, the fibers are parallel to each other. Note that the specimen orientation is not the same as in the actual window.

References

Chen, W.-R., Chen, S.-H. & Mallamace, F. (2002). *Physical Review E*, **66**(2), 021403.

Landman, J., Ouhajji, S., Prévost, S., Narayanan, T., Groenewold, J., Philipse, A. P., Kegel, W. K. & Petukhov, A. V. (2018). *Science Advances*, **4**(6), eaat1817.

Narayanan, T., Chèvremont, W. & Zinn, T. (2023). *Journal of Applied Crystallography*, **56**(4), 939–946.

Narayanan, T., Dattani, R., Möller, J. & Kwaśniewski, P. (2020). *Review of Scientific Instruments*, **91**(8), 085102.

Sztucki, M., Narayanan, T., Belina, G., Moussaid, A., Pignon, F. & Hoekstra, H. (2006). *Physical Review E*, **74**(5), 051504.
